# African traditional medicines for erectile dysfunction: elusive dream or imminent reality?

**Published:** 2007

**Authors:** John AO Ojewole

**Affiliations:** Department of Pharmacology, School of Pharmacy and Pharmacology, Faculty of Health Sciences, University of KwaZulu-Natal, Durban

Erectile dysfunction (ED) is a common medical condition that affects about 200 million men worldwide,^1^,^2^ and as many as 30 million American men.^3^ Although ED also afflicts many African men, authentic figures on the incidence and/or prevalence of the disease are not available in most African countries.

The National Institutes of Health (NIH) has defined erectile dysfunction as ‘a consistent inability (of the patient) to achieve and maintain erection sufficient for satisfactory sexual activity’.^3^ ED is associated with advancing age, with a 39% prevalence at the age of 40 years and 67% by 70 years of age.^4^ Erectile dysfunction is common in men with cardiovascular disorders such as ischaemic heart disease, hypertension and peripheral vascular diseases.^2^,^5^ It is also common in men with diabetes mellitus,^6^ probably because of the shared factors that impair haemodynamic mechanisms in the penile and ischaemic vasculature. Erectile dysfunction is also caused by spinal cord injury (cord level range, T6–L5),^7^ and other factors such as radical prostatectomy, long-term use of certain medications (eg, antidepressants, antipsychotics, antihypertensives and diuretics), indices of anger and depression, and cigarette smoking.^1^,^4^

Clinically known aetiologies of ED include organic, psychogenic and combined origins.^1^ Cardiovascular disorders and diabetes mellitus are known to contribute significantly to erectile dysfunction of organic origin.^6^,^8^ Organic causes of erectile dysfunction are found in 80–90% of ED patients, and these include vasculogenic (ie, arterial, cavernosal and mixed), neurogenic, anatomical and endocrine causes.4 Psychogenic forms of ED are usually due to sexual performance anxiety, depression and inhibited sexual drive.^4^

Recent studies have shown that vascular endothelial dysfunction is a major cause of ED, suggesting that ED might be an early manifestation of cardiovascular disease.^2^,^9^,^10^ Indeed, patients with ED possess many of the risk factors associated with coronary artery disease (CAD), such as smoking, hypertension, diabetes and hyperlipidaemia.^1^,^10^ The association between ED and CAD has, therefore, raised concerns regarding the cardiovascular safety of PDE-5 inhibitors.^10^

## Allopathic medicines for erectile dysfunction

Three synthetic drugs, namely sildenafil citrate (Viagra™), tadalafil (Cialis™) and vardenafil hydrochloride (Levitra™) are currently available on the pharmaceutical market for the treatment of ED. As a class, these compounds are mild vasoactive drugs and they act by selectively inhibiting the enzyme phosphodiesterase type-5 (PDE-5). PDE-5 catalyses the breakdown of the smooth muscle-relaxing agent, cyclic guanosine monophosphate (cGMP), a second messenger of nitric oxide.^10^ In the body, inhibition of PDE-5 increases cGMP levels, reduces intracellular calcium (Ca^2+^) and induces vasodilation.^10^,^11^ The drugs possess identical mechanisms of action, but differ essentially in their duration of action and in some aspects of their pharmacokinetic profiles. Recent clinical studies have shown that the three PDE-5 inhibitors are effective and relatively safe, and that they do not increase cardiovascular risk in patients with CAD.^12^-^14^

The availability of these PDE-5 inhibitors has provided effective and well-tolerated oral treatments for ED.^5^,^7^,^15^,^16^ Moreover, these drugs have been reported to improve endothelial function, 10,14 and are speculated to have vascular and myocardial protective properties.^10^,^17^ As a class, the three drugs are indicated for the treatment of erectile dysfunction only. They are contra-indicated in patients undergoing therapy with any form of nitrate, either regularly or intermittently. The common side effects of the three PDE-5 inhibitors include headache and dyspepsia, back pain, myalgia and non-arteritic anterior ischaemic optic neuropathy.

## Synthetic phosphodiesterase-5 inhibitors

Sildenafil citrate (Viagra™) and related sexual stimulant ‘love’ drugs have been widely studied for their tolerability, safety and efficacy in the treatment of erectile dysfunction in a variety of patient populations. In men, oral sildenafil citrate, tadalafil and vardenafil hydrochloride are generally known to be effective in erectile dysfunctions of organic, psychogenic or mixed origins. However, the aetiology of erectile dysfunction has been shown to have a significant impact on treatment success and satisfaction rates, with neurogenic causes of erectile dysfunction (eg, diabetes mellitus and prostate surgery) having significantly lower treatment success rates than psychogenic or vasculogenic erectile dysfunction.^15^

The pharmacokinetic characteristics of tadalafil differ significantly from those of sildenafil citrate and vardenafil hydrochloride. The mean half-life for both sildenafil and vardenafil is about four hours, whereas the mean half-life of tadalafil is 17½ hours, and tadalafil has also been shown to improve erectile dysfunction for up to 36 hours post dosing.^12^,^16^

## African traditional remedies for erectile dysfunction

In Africa, from ancient times, plants have served as a dependable and ever-ready source of medicines for the treatment of a plethora of chronic and acute diseases of mankind. The various communities and societies on the continent, in addition to ‘owning’ traditional remedies for ailments such as hypertension, diabetes mellitus, arthritis and other chronic conditions, also ‘own’ remedies for socio-cultural diseases such as erectile dysfunction.

Thousands of African medicinal plants (belonging to several genera and families, and with diverse chemical constituents) have been reported to possess aphrodisiac and sexual stimulant properties (Koloko, pers commun). Each African country has a catalogue of locally made, plant-derived sexual stimulants under various local trade names such as Impotex™, TigerPower™, SuperLove™, uBangalala™ and Burantashi™. Hundreds of such traditional, plant-derived remedies are used in African countries for the effective treatment of ED. For example, the Zulu people of South Africa have, for centuries, used the roots of Eriosema species as a remedy for the treatment of erectile dysfunction and/or impotence.

Generally, the genus *Eriosema* contains plants which fall under the Zulu indigenous umbrella name of *uBangalala*, and most of the plant species listed under this name are used mainly for the purpose of curing or alleviating impotence..^18^,^19^ Hot milk infusions of *Eriosema* roots and/or pounded boiled root decoctions of the plant are taken in small doses in the morning and at night for impotence.^18^,^19^ As with oral Viagra™ taken with a fatty meal, oral administration of an infusion or decoction of Eriosema roots with milk probably delays or reduces the rate of absorption of the bioactive compounds from the patient’s gastrointestinal tract, and thereby prolongs the duration of action of the compounds in the body.

It has been suggested that for maximum benefit, milk infusions and decoctions of *Eriosema kraussianum* roots are to be taken two to four hours before any anticipated sexual intercourse, and the effects (achievement and maintenance of penile erection sufficient for satisfactory sexual intercourse after penetration) of the plant extracts have been reported to last for four to six hours following oral dosing of the milk infusion or decoction of the rootstock extracts (Drewes, pers commun). Unlike Viagra™, however, the bioavailability, half-life, T_max_, C_max_ and other pharmacokinetic parameters of the bioactive compounds of *E kraussianum* are obscure at present. The effects of the extract on the biochemical activities of cGMP and PDE-5 are also unknown.

Due to economic constraints, providing adequate modern medical care to all the people in developing, third-world African countries is an elusive dream at present. Therefore, for the treatment of erectile dysfunction in the rural, peri-urban and some urban communities of Africa, it is prudent to look for salvation in herbal medicines and plant-derived products.

Recent studies in our laboratories^20^-^22^ have shown that *E kraussianum* NE Br (Fabaceae) is one of the promising plant species of South Africa with potential for use as an effective remedy in the treatment of impotence and/or ED. Drewes *et al.*^20^,^21^ have shown, in a rabbit experimental model, the plausible therapeutic beneficial effects of bioactive compounds of *E kraussianum* in the management of erectile dysfunction. The psycho-social benefits of using such plant-derived crude remedies in rural African communities cannot be overemphasised. Since men with ED of organic, psychogenic and mixed aetiologies are known to benefit from Viagra™ therapy, it is speculated that *E kraussianum* extractives may also be used effectively as a Viagra™ substitute in South African men with such erectile dysfunction.

## Conclusion

There is a dire need to develop some of the existing potent, African traditional remedies for erectile dysfunction into scientifically acceptable natural medicines. With the financial and goodwill support of governments, non-governmental organisations and philanthropic individuals, coupled with the cooperation of multinational pharmaceutical companies such as Pfizer and others, it should be possible to develop some of the currently available African traditional remedies for ED into acceptable, potent natural medicines in the foreseeable future. Such existing remedies should be subjected to rigorous scientific scrutiny experimentally (in laboratory animals) and clinically (in humans), in order to establish their safety, efficacy, quality, mechanisms of action, side effects, and possibly also, their contra-indications.

The goals of medicines, whether allopathic, traditional or complementary, are the same, namely, to benefit patients therapeutically and improve their quality of life. Based on these assumptions, one can look forward to a near future of integrated orthodox and traditional medicines, and hope that experimental and clinical research in traditional, complementary and alternative medicines will help to develop affordable, safe and effective natural medicines for erectile dysfunction, rather than criticising and marginalising unorthodox medicines, ethnomedical claims and traditional findings.

With traditional health practitioners, pharmacists, orthodox medical practitioners, nurses, botanists, chemists, pharmacologists, toxicologists and other scientists working together collaboratively for a common purpose, the future of scientifically developed, affordable, safe and effective natural medicines for ED will certainly be in sight. Now is the time to ensure that future availability of scientifically formulated, safe and effective traditional medicines for the treatment of erectile dysfunction is not an elusive dream, but an imminent reality.
